# Role of *Chitin Deacetylase 1* in the Molting and Metamorphosis of the Cigarette Beetle *Lasioderma serricorne*

**DOI:** 10.3390/ijms21072449

**Published:** 2020-04-01

**Authors:** Wen-Jia Yang, Kang-Kang Xu, Yi Yan, Can Li, Dao-Chao Jin

**Affiliations:** 1 Provincial Key Laboratory for Agricultural Pest Management of Mountainous Regions, Institute of Entomology, Guizhou University, Guiyang 550025, China; yangwenjia1985@gmail.com (W.-J.Y.); kkxu1988@gmail.com (K.-K.X.); yanheyi95@gmail.com (Y.Y.); 2 Guizhou Provincial Key Laboratory for Rare Animal and Economic Insect of the Mountainous Region, College of Biology and Environmental Engineering, Guiyang University, Guiyang 550005, China

**Keywords:** chitin deacetylase, cigarette beetle, molting, wing development, RNA interference

## Abstract

Chitin deacetylases (CDAs) are chitin-modifying enzymes known to play vital roles in insect metamorphosis and development. In this study, we identified and characterized a *chitin deacetylase* *1* gene (*LsCDA1*) from the cigarette beetle *Lasioderma serricorne*. *LsCDA1* contains a 1614 bp open reading frame encoding a protein of 537 amino acids that includes domain structures typical of CDAs. *LsCDA1* was mainly expressed in the late larval and late pupal stages. In larval tissues, the highest level of *LsCDA1* was detected in the integument. The expression of *LsCDA1* was induced by 20-hydroxyecdysone (20E) in vivo, and it was significantly suppressed by knocking down the expression of ecdysteroidogenesis genes and 20E signaling genes. RNA interference (RNAi)-aided silencing of *LsCDA1* in fifth-instar larvae prevented the larval–pupal molt and caused 75% larval mortality. In the late pupal stage, depletion of *LsCDA1* resulted in the inhibition of pupal growth and wing abnormalities, and the expression levels of four wing development-related genes (*LsDY*, *LsWG*, *LsVG*, and *LsAP*) were dramatically decreased. Meanwhile, the chitin contents of *LsCDA1* RNAi beetles were significantly reduced, and expressions of three chitin synthesis pathway genes (*LsTRE1*, *LsUAP1*, and *LsCHS1*) were greatly decreased. The results suggest that *LsCDA1* is indispensable for larval–pupal and pupal–adult molts, and that it is a potential target for the RNAi-based control of *L. serricorne*.

## 1. Introduction

Molting and metamorphosis are important aspects of insect growth, as the insect periodically sheds and replaces the rigid exoskeleton. The insect cuticle is repeatedly renewed while the old cuticle is digested, and a series of enzymes and cofactors function in the process of molting and development [1,2]. Chitin is a linear polymer of *N*-acetylglucosamine and is a major structural component of the insect cuticle [3]. Chitin synthesis and degradation are tightly coupled with the chitin content balance, which is key for the molting cycle and metamorphosis of insects [4,5]. Chitin degradation is accomplished by three types of chitinolytic enzyme: chitin deacetylases (EC 3.5.1.41, CDAs), chitinase (EC 3.2.1.14, CHTs), and *β*-*N*-acetylglucosaminidases (EC3.2.1.30, NAGs). CDAs enzymatically alter chitin by a deacetylating process, whereas CHTs and NAGs degrade chitin by hydrolyzation [6,7]. These three enzymes mainly function in insect growth and development. Studies in *Nilaparvata lugens* revealed that one *NAG*, five *CHTs*, and three *CDAs* participate in chitin degradation and are critical for nymph molting [8,9,10].

CDAs are extracellular chitin-modifying enzymes and belong to the carbohydrate esterase family 4 [11]. The first cDNA encoding chitin deacetylase-like protein was cloned from the cabbage looper *Trichopusia ni* [12]. Since then, hundreds of CDA genes have been identified in numerous insect species, including Coleoptera [13,14,15,16], Hemiptera [10], Hymenoptera [17], Orthoptera [18,19], Diptera [20,21], Lepidoptera [7,22,23,24,25], and Isoptera [26]. Based on sequence similarity and domain organization, insect CDAs have been divided into five groups (group I−V) [17]. Both group I and II CDAs contain a chitin-binding (ChtB) domain, a low-density lipoprotein receptor class A (LDLa) domain, and a polysaccharide deacetylase-like (CDA) catalytic domain, but with substantial differences in amino acid sequences. Group III and IV CDAs contain ChtB and CDA catalytic domains, whereas group V CDA has only the CDA catalytic domain [17]. Different groups of CDA have various functions during insect development. For example, studies have been conducted in the model species *Tribolium castaneum*, in which all nine *CDA* genes have been identified and characterized. The RNA interference (RNAi)-mediated knockdown of *TcCDA1* or *TcCDA2* resulted in severe molt blocks at the larval–larval, larval–pupal, and pupal–adult transitions; however, no developmental abnormalities were observed after the RNAi knockdown of *TcCDA3* to *TcCDA9* [13]. Based on the complete genome sequencing of *N. lugens*, four *CDA-like* genes were also identified. The silencing of *NlCDA1*, *NlCDA2*, and *NlCDA4* caused molting failure and high mortality, while nonviable phenotypes were found after injection with double-stranded RNA (dsRNA) for *NlCDA3* [10]. Similar molting defects were observed after RNAi of *CDA* genes in many insect species, including *Stegobium paniceum* [14], *Leptinotarsa decemlineata* [15,16], *Locusta migratoria* [19], *Choristoneura fumiferana* [22], *Hyphantria cunea* [24], *Bombyx mori* [25], and *Heortia vitessoides* [27]. In *Drosophila melanogaster*, mutations of *DmCDA1* (*Serpentine*) and *DmCDA2* (*vermiform*) led to limited tracheal elongation and affected the formation of the larval cuticle [20,28,29]. Subsequently, RNAi experiments confirmed that *DmCDA1* and *DmCDA2* have distinct roles in *Drosophila* adult wing development [30]. Some *CDAs* in *Bombyx mori* and *Mamestra configurata* were found to be expressed mainly in the peritrophic membrane (PM) and to play important roles in modifying the physical properties of the PM [31,32].

The cigarette beetle *Lasioderma serricorne* (Fabricius) (Coleoptera: Anobiidae) is a destructive and widespread storage pest that feeds on postharvest agricultural products, such as grains, tobacco leaves, spices, dry foods, and Chinese medicinal materials [33,34]. Adult beetles do not feed; they simply locate suitable products, mate, and lay eggs [35]. Immediately after egg hatching, *L. serricorne* larvae directly feed on the stored products and cause serious economic damage [36]. Many methods have been employed for *L. serricorne* management, including using extreme temperatures [37,38], a low-oxygen modified atmosphere [39], entomopathogenic fungi [40], and ultraviolet light [41]. The most effective control methods rely heavily on the use of insecticides and repellents, such as phosphine [42,43], pyrethrin [44], and essential oils [45]. Unfortunately, overreliance on chemical controls has led to several problems such as insecticide resistance, environmental pollution, and pest resurgence [42,43]. There is an urgent need to find novel and sustainable control strategies. Due to chitin being absent in plants and vertebrates, chitin metabolic enzymes have been considered as potential targets for eco-friendly insecticide development [46]. Recently, we identified two chitin-degrading enzyme genes (*LsNAG1* and *LsNAG2*), and demonstrated their crucial roles in *L. serricorne* molting and wing development [47,48]. In this study, we report: (1) the full-length open reading frame (ORF) of the *chitin deacetylase 1* gene (*LsCDA1*) in *L. serricorne*, (2) the expression profiles of *LsCDA1* in different developmental stages and larval tissues, (3) the effects of 20-hydroxyecdysone (20E) and the 20E signaling pathway on the expression of *LsCDA1* in the fourth-instar larvae of *L. serricorne*, and (4) the functional analysis of *LsCDA1* by RNAi in the larval–pupal and pupal–adult transitions. These analyses will facilitate our understanding of the potential functions of *LsCDA1* in *L. serricorne* development.

## 2. Results

### 2.1. Identification and Characterization of LsCDA1

**Figure 1:**
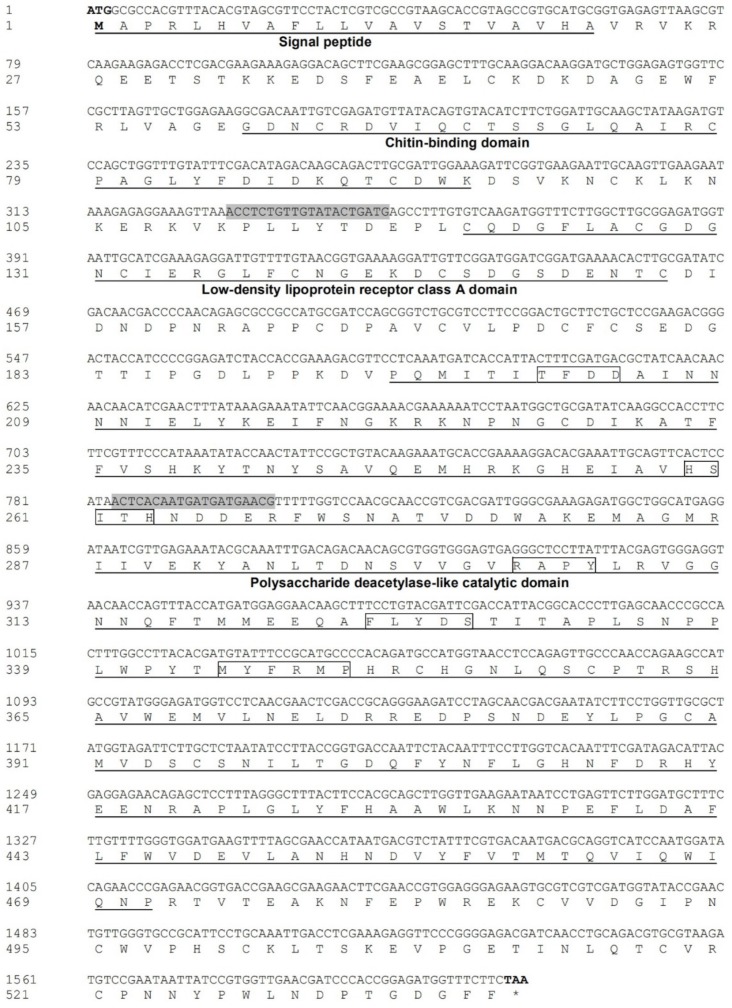
The nucleotide and deduced amino acid sequences of *LsCDA1* cDNA. The start codon is indicated in bold and the stop codon in bold with an asterisk. The predicted signal peptide, chitin-binding domain, low-density lipoprotein receptor class A domain, and polysaccharide deacetylase-like catalytic domain are underlined. Five signature motifs (motifs 1–5) of the sequence catalytic domain are boxed. The primers for *LsCDA1* dsRNA synthesis are shaded.

Based on the *L. serricorne* transcriptome, the putative cDNA sequence of *LsCDA1* (GenBank accession number AXU05961.1) was obtained and verified by reverse transcription PCR (RT-PCR). The ORF of *LsCDA1* consisted of 1614 bp, encoding 537 amino acid residues with a calculated molecular weight of approximately 61.1 kDa and a theoretical isoelectric point of 4.99. Signal peptides with 21 amino acids (from M1 to A21) were found at the N-terminal ends of LsCDA1. The deduced amino acid sequence of *LsCDA1* includes a putative ChtB domain (residues 59–94), a LdLa domain (residues 120–154), and a CDA catalytic domain (residues 195–471). In the CDA catalytic region of the LsCDA1 protein, five signature motifs (TFDD, HSITH, RAPY, FLYDS, and MYFRMP) characteristic of the deacetylase proteins were also identified (Figure 1).

**Figure 2:**
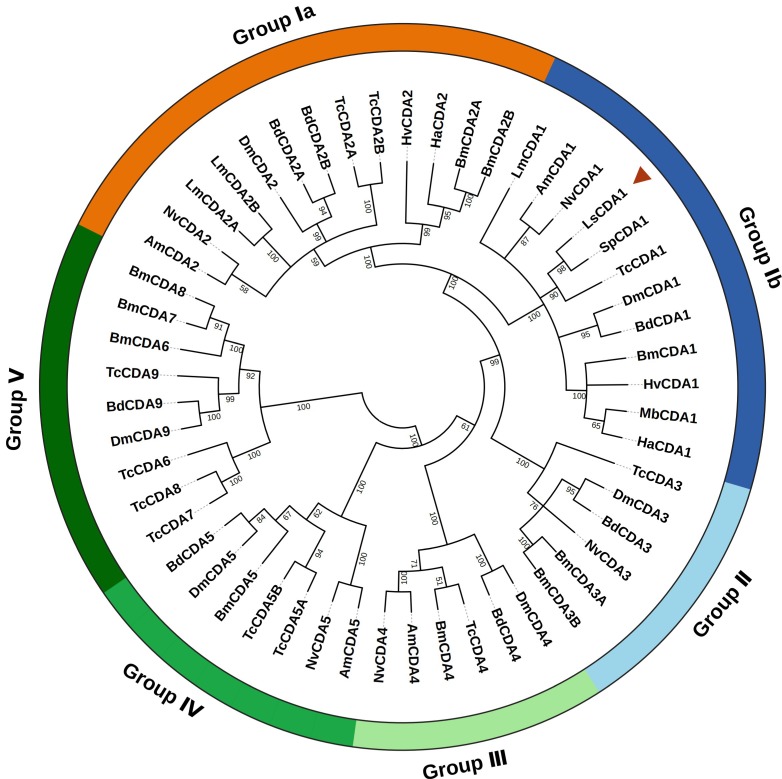
Phylogenetic analysis of insect chitin deacetylases (CDAs). The phylogenetic tree was constructed using the MEGA7 software based on the Maximum Likelihood method. A bootstrap analysis of 1000 replications was used, and bootstrap values (only above 50%) are shown in the cladogram. Am, *Apis mellifera*; Bd, *Bactrocera dorsalis*; Bm, *Bombyx mori*; Dm, *Drosophila melanogaster*; Ha, *Helicoverpa armigera*; Hv, *Heortia vitessoides*; Ld, *Leptinotarsa decemlineata*; Lm, *Locusta migratoria*; Ls, *Lasioderma serricorne*; Mb, *Mamestra brassicae*; Nv, *Nasonia vitripennis*; Sp, *Stegobium paniceum*; Tc, *Tribolium castaneum*. The *L. serricorne* LsCDA1 protein is marked with a red triangle. The sequences presented in the phylogenetic tree are listed in Supplementary Table S1.

Sequence alignment showed that LsCDA1 shared 96.5%, 93.4%, 91.3%, and 88.5% identity with the CDA1 of *S. paniceum* (AYA83838.1), *T. castaneum* (NP_001095946.1), *L. migratoria* (ANA57443.1), and *Ostrinia furnacalis* (AKJ26157.1), respectively. Phylogenetic analysis was performed to display the relationships among these insect CDAs. The tree showed that these proteins were divided into five major groups (I–V); LsCDA1 was most closely related to SpCDA1 of *S. paniceum* and belonged to group Ib CDAs (Figure 2).

### 2.2. Developmental and Tissue-Specific Expression of LsCDA1

**Figure 3:**
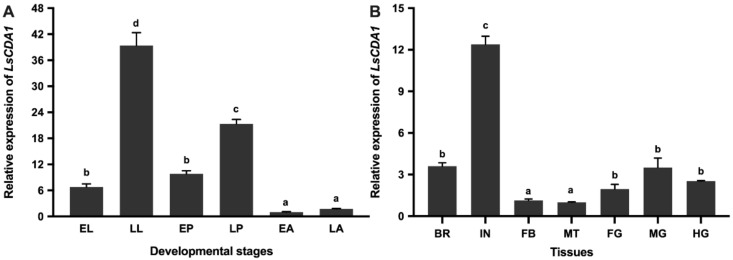
Expression profiles of *LsCDA1* in the different developmental stages (**A**) and different larval tissues (**B**) of *L. serricorne*. Various stages are collected. EL, early larvae; LL, late larvae; EP, early pupae; LP, late pupae; EA, early adults; LA, late adults. Different tissues are listed. BA, brain; IN, integument; FB, fat body; MT, Malpighian tubules; FG, foregut; MG, midgut; HG, hindgut. Data were normalized to the geometric mean of the expression of *EF1α* and *18S*. Different letters above bars indicate significant differences based on one-way ANOVA followed by a least significance difference test (*p* < 0.05).

Quantitative real-time PCR (qPCR) was used to analyze the expression of *LsCDA1* in different developmental stages and different tissues of *L. serricorne*. Among the six assessed stages, *LsCDA1* was stably expressed at all of the stages; however, it was highly expressed in the late larval and late pupal stages (Figure 3a). The expression of *LsCDA1* varied among the seven tissues of late larvae, and the highest expression level of *LsCDA1* was detected in the integument (Figure 3b).

### 2.3. Expression of LsCDA1 in Response to 20E Signaling

**Figure 4:**
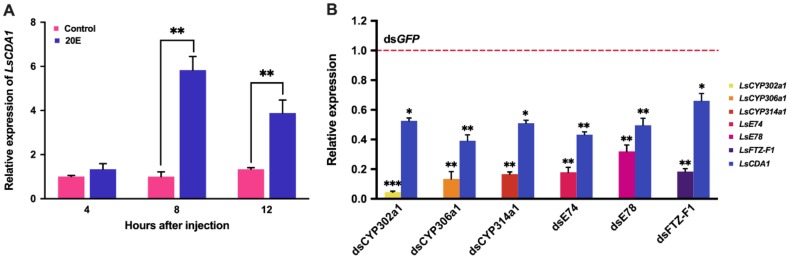
20-Hydroxyecdysone-responsiveness of *LsCDA1* in *L. serricorne*. (**A**) The in vivo effects of 20E on the expression of *LsCDA1*. The larvae were collected for qPCR analysis at 4, 8 and 12 h after injection with 20E. Control: insects injected with distilled water containing 0.1% ethanol; 20E: insects injected with 20E (120 ng/larva). (**B**) The relative expression levels of *LsCDA1*, *LsCYP302a1*, *LsCYP306a1*, *LsCYP314a1*, *LsE74*, *LsE78*, and *LsFTZ-F1* at 3 days after gene-specific dsRNA injection. The gene expression in the ds*GFP* group was set as 1 and is shown by the dotted line. Data were normalized to the geometric mean of the expression of *EF1α* and *18S*. Significant differences between the treatment group and control group were determined using Student’s *t*-test (* *p* < 0.05, ** *p* < 0.01, *** *p* < 0.001).

To test whether *LsCDA1* is induced by 20E, the fourth-instar larvae were injected with 20E for 4, 8 and 12 h. The expression of *LsCDA1* in the 20E treatment group was significantly increased, and the ratios of inducible expression were 5.8- and 3.9-fold at 8 and 12 h post-injection, respectively, compared to the control group (Figure 4a). To further validate the regulation of *LsCDA1* in response to 20E signaling, we individually silenced genes encoding ecdysteroidogenesis enzymes (*CYP302a1*, *CYP306a1*, and *CYP314a1*) and 20E signaling components (*E74*, *E78*, and *FTZ-F1*) by the injection of dsRNAs. The mRNA level of *LsCDA1* was significantly decreased in the *CYP302a1*, *CYP306a1*, and *CYP314a1* RNAi larvae compared to that in controls. We also found that the inhibition of the 20E signal in the *E74*, *E78*, and *FTZ-F1* RNAi larvae significantly downregulated the expression of *LsCDA1* (Figure 4b).

### 2.4. Knockdown of LsCDA1 Impairs Larval–Pupal and Pupal–Adult Transitions

**Figure 5:**
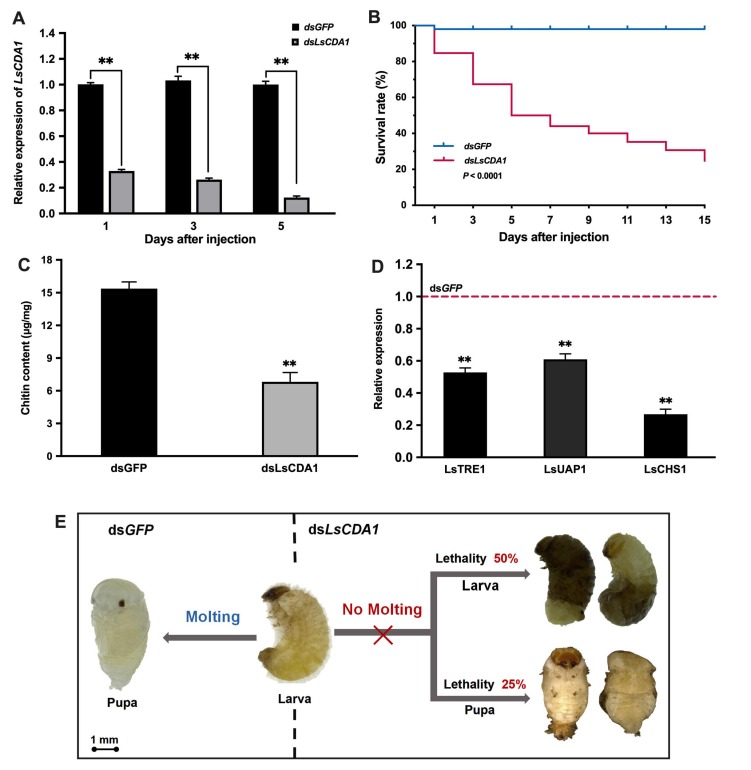
The effect of *LsCDA1* knockdown on larval–pupal molting in *L. serricorne*. (**A**) The relative expression levels of *LsCDA1* at 1, 3, and 5 days after *LsCDA1* or *GFP* dsRNA injection in the fifth-instar larvae. (**B**) Kaplan-Meier survival curves of *L. serricorne* larvae after *LsCDA1* or *GFP* dsRNA injection. The effects of *LsCDA1* knockdown on chitin contents (**C**) and the expressions of three chitin synthesis pathway genes (**D**). The expression values of chitin synthesis pathway genes were calculated by comparison to the ds*GFP* group, which was normalized at 1. (**E**) Representative phenotypes of larvae after *LsCDA1* or *GFP* dsRNA injection. Significant differences between the RNAi group and the control group were determined using Student’s *t*-test (** *p* < 0.01).

To investigate the role of *LsCDA1* in the molting process of *L. serricorne,* dsRNAs of *LsCDA1* and *green fluorescent protein* (*GFP)* were synthesized in vitro and injected into fifth-instar larvae and late pupae, respectively. Compared to the control, the expression of *LsCDA1* was significantly reduced after injection with ds*LsCDA1* in the fifth-instar larvae (Figure 5a). The survival rate of *L. serricorne* was reduced to 25% in the ds*LsCDA1* group, whereas 98% of the individuals in the control group could molt normally to pupae in the ds*GFP* group (Figure 5b). After the silencing of *LsCDA1*, the chitin contents were greatly decreased in the *LsCDA1* RNAi larvae compared to those in the control samples (Figure 5c). The expression levels of chitin synthesis pathway genes, *trehalase 1* (*LsTRE1)*, *UDP*-*N*-*acetylglucosamine pyrophosphorylase 1* (*LsUAP1),* and *chitin synthase 1* (*LsCHS1*) were significantly lowered in the *LsCDA1*-depleted larvae (Figure 5d). Following the injection of ds*LsCDA1*, 50% of larvae were unable to shed their old cuticles and failed to complete the larval–pupal molting, and they eventually died; approximately 25% of the individuals successfully molted but died later (Figure 5e).

**Figure 6:**
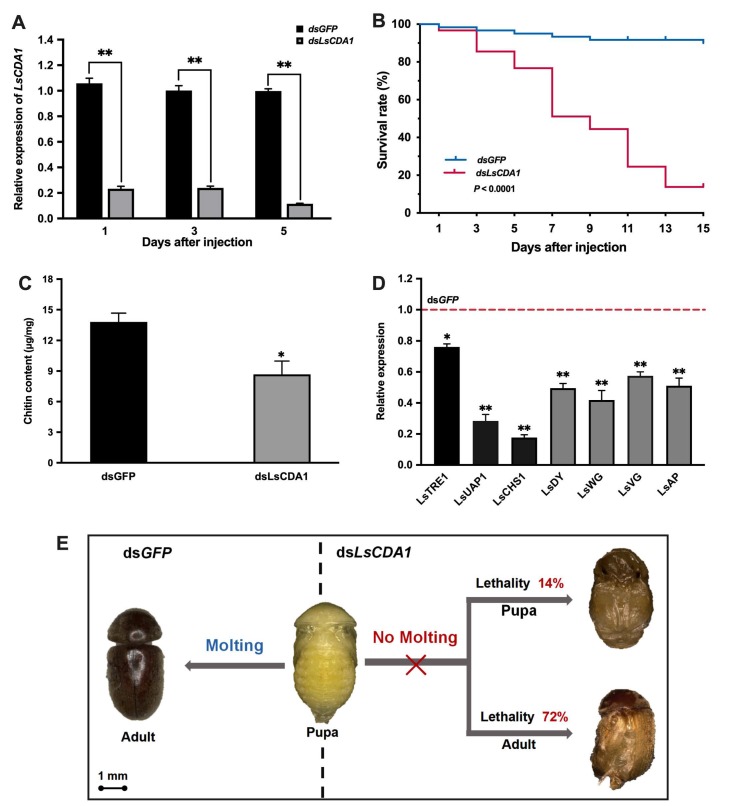
The effect of *LsCDA1* knockdown on pupal–adult metamorphosis in *L. serricorne*. (**A**) The relative expression levels of *LsCDA1* at 1, 3, and 5 days after *LsCDA1* or *GFP* dsRNA injection at the late pupal stage. (**B**) Kaplan-Meier survival curves of *L. serricorne* pupae after *LsCDA1* or *GFP* dsRNA injection. The effects of *LsCDA1* knockdown on chitin contents (**C**) and the expressions of three chitin synthesis pathway genes and four genes involved in wing development (**D**). The expression values were calculated by comparison to the ds*GFP* group, which was normalized at 1. (**E**) Representative phenotypes of pupae after *LsCDA1* or *GFP* dsRNA injection. Significant differences between the RNAi group and the control group were determined using by Student’s *t*-test (* *p* < 0.05, ** *p* < 0.01).

For the late pupae treated with ds*LsCDA1*, the transcript level of *LsCDA1* was significantly decreased compared with the ds*GFP* group (Figure 6a). The survival rate of pupae injected with ds*LsCDA1* was significantly lower compared to the controls, and 86% of individuals exhibited abnormal phenotypes and eventually died during the pupal–adult transition (Figure 6b). The chitin contents of the pupae were also reduced after the injection of ds*LsCDA1* compared to those of control insects (Figure 6c), and the expression levels of three chitin synthesis pathway genes were significantly reduced in *LsCDA1* RNAi pupae (Figure 6d). Furthermore, we examined the expression of four genes related to wing development in pupae after *LsCDA1* silencing. The results showed that the expression levels of four genes, *wingless* (*LsWG*), *vestigial* (*LsVG*), *dusky* (*LsDY*), and *apterous* (*LsAP*), were significantly decreased compared to those in control insects (Figure 6d). Specifically, 14% of pupae failed to shed puparia and died without completing adult eclosion, whereas 72% of the pupae molted to deformed adults and emerged with wrinkled wings (Figure 6e). No visible phenotypic changes were observed in the ds*GFP*-injected group.

## 3. Discussion

### 3.1. Characteristics and Expression Profiles of LsCDA1

Chitin deacetylases play essential roles in chitin degradation during insect growth and development, and these enzymes have been identified in several insect species [10,13,14,15,16]. In this study, we report the complete ORF sequence encoding chitin deacetylase 1, *LsCDA1*, from the cigarette beetle. Bioinformatic analysis showed that LsCDA1 contained a signal peptide, a ChtB domain, an LDLa binding domain, and a CDA catalytic domain, indicating that it belonged to the carbohydrate esterase family 4 [11]. These structural domains are also considered as expression sequence tags of group I and II chitin deacetylases [7,17]. Five well-conserved motifs of deacetylase proteins [49], TFDD, HSITH, RAPY, FLYDS, and MYFRMP, were present in the CDA catalytic region of LsCDA1. Homology alignment and phylogenetic analysis revealed that LsCDA1 belonged to subgroup Ib CDAs. LsCDA1 shares a closer relationship with SpCDA1 in *S. paniceum* and TcCDA1 in *T. castaneum*, suggesting that they may have similar functions during insect molting [13,14].

Developmental expression analyses indicated that *LsCDA1* was highly expressed in late larval and late pupal stages, which are specific periods prior to each molt. Similar results have been confirmed for many *CDA1* genes of other insect species, such as *N. lugens* [10], *T. castaneum* [13], *S. paniceum* [14], and *H. vitessoides* [27]. The dramatically increased expression of *CDA1* was probably related to the requirement for chitin deposition during molt periods. Our previous studies have demonstrated a significant increase in the expression levels of two chitin-degrading enzyme genes (*LsNAG1* and *LsNAG2*) during the larval–pupal and pupal–adult molts of *L. serricorne* [47,48]. Collectively, these results suggest that *LsCDA1* and two *LsNAGs* signified their important roles in the molting of *L. serricorne*. In *Helicoverpa armigera*, large amounts of *HaCDA1* transcripts were observed in the fat body [23]. In *D. melanogatser*, *DmCDA1* was highly expressed in the embryonic trachea [28]. In *L. decemlineata*, *LdCDA1* was copiously expressed in the larval ileum, rectum, and epidermis [15]. In *L. migratoria*, *LmCDA1* was highly expressed in the foregut, and lower transcripts were also detected in the integument and hindgut, but were not detected in other examined tissues [19]. The rice leaf folder *CmCDA1* was expressed primarily in the head and midgut [50]. In the pharate appendages of *T. castaneum* adults, *TcCDA1*-specific in situ hybridization showed a strong signal in the epidermal cells [13]. In the present study, *LsCDA1* was predominantly expressed in the integument at the larval stages, which is consistent with the expression of *NlCDA1*, *NlCDA2*, and *NlCDA4* from *N. lugens* [10], *HcCDA2* from *H. cunea* [24], and *BmCDA1* and *BmCDA2* from *B. mori* [25]. We speculated that these CDAs are responsible for chitin metabolism in the integument and are crucial for insect molting. Actually, the results were not always in accordance with those of previous studies. For example, the injection of dsRNAs of *BmCDA1* and *BmCDA2* in the larvae of *B. mori* did not reveal any morphological abnormalities, but rather led to delayed pupation times [25]. Therefore, we proposed that the high expression of *CDAs* in the integument might be associated with additional physiological functions.

### 3.2. Transcriptional Regulation of LsCDA1 by 20E

There is a functional relationship between 20E and the chitin metabolism system. It has been documented that 20E and its signal pathway play vital roles in regulating insect molting and metamorphosis [51]. Cholesterol or other sterols are precursors and are converted to ecdysone and its derivative 20E by a series of *Halloween* genes, such as *Spook* (*CYP307a1*), *Phantom* (*CYP306a1*), *Disembodied* (*CYP302a1*), *Shadow* (*CYP315a1*), and *Shade* (*CYP314a1*) [52]. Then, 20E binds its complex nuclear receptors, and transduces its signal through a subset of primary response genes (*E74*, *E75*, *Broad*, *HR3*, *HR4*, *E78*, *FTZ-F1*, and others) [53]. Previous studies in *Spodotera exigua* showed that 20E and its receptors could induce the expression of five chitin synthesis pathway genes [54]. In *L. migratoria*, the injection of 20E and the downregulation of ecdysone receptor genes increased and decreased the expression of two variants of the *chitinase 5* gene (*LmCht5*) [55]. The injection of 20E could upregulate the gene expression of *NAG* genes in *L. serricorne* [47] and *L. migratoria* [56]. Interestingly, 20E also stimulated the expression of the *chitin synthase 1* gene in *B. dorsalis* [57], *B. mori* [58], and *L. decemlineate* [59]. In this study, we found that *LsCDA1* expression was induced by 20E in vivo, and the expression was suppressed by the silencing of three ecdysteroidogenesis genes (*CYP302a1*, *CYP306a1*, and *CYP314a1*) and three 20E signaling genes (*E74*, *E78*, and *FTZ-F1*). The results indicated that *LsCDA1* expression could be regulated by 20E synthesis and the 20E signaling pathway. We confirmed that *LsCDA1* was affected by a 20E pulse. Similar findings have been reported for *LdCDA1* and *LdCDA2* in *L. decemlineata*; however, the expressions levels of two *LdCDAs* were inhibited after the application of juvenile hormone (JH) and its analogs, and were stimulated by the silencing of JH synthesis and signaling genes [15,16]. In *S. paniceum* [14] and *H. vitessoides* [27], exposure to 20E could activate the expression of *CDA* genes at various times. Substantial upregulation of six *BmCDAs* (*BmCDA1*–*BmCDA6*) by 20E was observed not only in the larval cuticles and midguts, but also in embryo cell lines of *B. mori* [25]. These results strongly suggest that CDAs are important 20E late-responsive genes. 

### 3.3. Knockdown of LsCDA1 Affects Chitin Metabolism and Impairs Molting

It has been reported that *CDA1* plays roles in the formation of the cuticle during insect molting. In this study, RNAi was used to evaluate the functions of *LsCDA1*, and specific ds*LsCDA1* was injected into the fifth-instar larvae and late pupae of *L. serricorne*. Our results revealed that the silencing of *LsCDA1* caused developmental disturbances and lethal morphological phenotypes, suggesting that *LsCDA1* is critical for successful larval–pupal and pupal–adult molts. This phenomenon is consistent with previous reports. In *T. castaneum* and *N. lugens*, the knockdown of *CDA1* disrupted the metamorphic transitions, and the treated insects failed to shed their old cuticles and eventually died [10,13]. In *Oxya chinensis*, nymphs injected with ds*CDA1* displayed arrested development, and more than 95% died during the molting process [18]. The function of *CDA1* in the molting was also studied in *S. paniceum* [14], *L. migratoria* [19], and *H. vitessoides* [27]. Given that CDA1 is essential for insect metamorphosis, it could be a promising target for RNAi-based insecticide development. Meanwhile, we found the chitin content was significantly reduced in the *LsCDA1* RNAi beetles, and the expression levels of *LsTRE1*, *LsUAP1*, and *LsCHS1* were dramatically decreased. Similar results have been observed in *L. decemlineata* larvae, where RNAi of *LdCDA1* decreased the chitin amounts and the mRNA levels of five chitin synthesis pathway genes [15]. This indicates that repression of *CDA1* may simultaneously affect chitin synthesis during the molting process. Taken together, the results suggest that *LsCDA1* is responsible for chitin degradation and synthesis in the cuticle, and is critical for *L. serricorne* development. However, the expression levels of chitin synthesis pathway genes were not affected after the silencing of *HvNAG1* in the larvae of *H. vitessoides* [60]. Therefore, the underlying mechanism of how chitin-degrading enzymes assist chitin synthesis is complicated and needs further investigation.

### 3.4. The Function of LsCDA1 Involved in Wing Development During Pupal–Adult Metamorphosis

Functional studies in *D. melanogaster* demonstrated that Group I CDAs play distinct roles in wing development. Specifically, *DmCDA1* is the main enzyme responsible for chitin deacetylation in wing cuticle formation, whereas *DmCDA2* is required for the laminar arrangement of chitin. Additionally, both enzymes contribute to the establishment of the cuticular inward barrier against the penetration of xenobiotics [30]. In the current study, the silencing of *LsCDA1* in the late pupae of *L. serricorne* resulted in wing deformities. In this group, 72% of the individuals molted to adults but died with wrinkled wings. Similar phenotypes have also been observed after RNAi of *CDA1* in *L. decemlineata* [15] and *H. vitessoides* [27]. Interestingly, other chitin-degrading enzymes, such as NAG and chitinase, have been reported to be crucial for normal wing formation. In *H. vitessoides* and *L. serricorne*, the suppression of *NAG* expression led to abnormal wing phenotypes [47,60]. In *Sogatella furcifera*, the injection of *SfCHT7* dsRNA caused individuals to emerge with deformed wings [61]. It seems that chitin deposition is indispensable for adult wing development. However, the mechanism by which chitin-degrading genes control the development of wings remains unclear. Previous studies demonstrated that these genes, such as *dusky* (*DY*), *wingless* (*WG*), *vestigial* (*VG*), and *apterous* (*AP*), have essential roles during the wing morphogenesis of *T. castaneum* [62,63]. Here, we found that the expression levels of *LsDY*, *LsWG*, *LsVG*, and *LsAP* were downregulated after the knockdown of *LsCDA1* in *L. serricorne*. This suggests that *LsCDA1* controls beetle molting by regulating the expression of wing development genes during the pupal–adult transition.

## 4. Materials and Methods

### 4.1. Insects

The cigarette beetle *L. serricorne* was originally collected from a tobacco warehouse (N26°30′, E106°40′) in Guizhou Province, China. All of the insects were incubated in our laboratory at 28 °C under a constant 24-h darkness with 40% relative humidity. The larvae were reared on the dried roots of *Angelica sinensis* as described previously [47].

### 4.2. Molecular Cloning and Bioinformatic Analysis

Total RNA was extracted from *L. serricorne* larvae using a MiniBEST Universal Extraction Kit (TaKaRa, Dalian, China), following the manufacturer’s instructions. The integrity and quantity of RNA were evaluated using 1% agarose gel electrophoresis and a NanoDrop 2000C spectrophotometer (Thermo Fisher Scientific, Waltham, MA, USA). One microgram of total RNA was used to synthesize first-strand cDNA with the TransScript Synthesis Supermix (TransGen, Beijing, China). The cDNA library of *L. serricorne* integument was constructed using an mRNA sequencing assay on an Illumina HisSeq2000 platform by Biomarker Technologies (Beijing, China). The putative *CDA1* sequence was obtained from this transcriptome database. The full-length ORF sequence of *CDA1* was acquired by using the ORF Finder software (https://www.ncbi.nlm.nih.gov/orffinder). The correctness of the sequence was substantiated by RT-PCR using specific primers (Supplementary Table S2). PCR was conducted as follows: 95 °C for 5 min; 34 cycles of 95 °C for 30 s, 57 °C for 30 s, and 72 °C for 2 min; and then an extension at 72 °C for 10 min. The PCR product was cloned into the pMD19-T vector (TaKaRa, Dalian, China) and then sequenced (Tsingke Bio, Chengdu, China).

Sequence similarities were determined using the Basic Local Alignment Search Tool program of the NCBI website (https://blast.ncbi.nlm.nih.gov/Blast.cgi). DNAMAN 6.0 (LynnonBiosoft, Vaudreuil, Quebec, Canada) was used to edit the sequence. The signal peptide, molecular weight, and isoelectric point were predicted by the ExPASy Proteomics Server (http://www.expasy.ch). Domain predictions were produced by the Simple Modular Architecture Research Tool (http://smart.embl.de/). Amino acid sequences were aligned with Clustal W [64]. Phylogenetic tree construction was performed using MEGA7 [65] with the Maximum Likelihood method and General Reversible Chloroplast model with 1000 bootstrap replicates.

### 4.3. Developmental and Tissue-Specific Expression Analysis of LsCDA1

Developmental samples were collected from early larvae (<24 h after hatching), late larvae (older than fourth-instar, including prepupae), early pupae (>48 h after pupation), late pupae (>120 h after pupation, before emergence), early adults (<24 h after emergence), and late adults (one week old). For tissue distribution, seven tissues (integument, brain, foregut, midgut, hindgut, fat body, and Malpighian tubules) were dissected from the late larvae. Each sample included 30–50 individuals, and three biological replications were prepared. Total RNA extraction and first-strand cDNA synthesis were performed as described above. A qPCR was used to detect the expression profiles of *LsCDA1*. The qPCR was conducted on a CFX-96 real-time PCR Detection system (Bio-Rad, Hercules, CA, USA) with the GoTaq qPCR Master Mix (Promega, Madison, WI, USA) under the following conditions: initial denaturation at 95 °C for 2 min, followed by 40 cycles 95 °C for 30 s and 60 °C for 30 s. A melting curve analysis was performed to ensure the specificity of the qPCR. The fivefold dilution series of cDNA from the late larvae was used to construct a relative standard curve to determine the PCR efficiency of each primer for each gene. *L. serricorne* elongation factor 1-alpha (*EF1α*) and 18S ribosomal RNA (*18S*) were used as internal reference genes according to our published results [48,66]. Data were analyzed by the 2^−∆∆Ct^ method [67], using the geometric mean of the two internal reference genes for normalization. All methods and data were confirmed to follow the Minimum Information for Publication of Quantitative Real-Time PCR Experiments (MIQE) guidelines [68].

### 4.4. Expression of LsCDA1 after 20E Treatment and RNAi of Ecdysone Synthesis and Signaling Genes

The newly ecdysed fourth-instar larvae were used for 20E treatment. According to our previous study [47], each larva in the treatment group was injected with 120 ng of 20E (Sigma-Aldrich, St. Louis, MO, USA) dissolved in 0.1% ethanol, to a final concentration of 1 μg/μL, while the control group was injected with an equal volume of 0.1% ethanol. Each larva was injected at the junction between the second and third abdominal segments using a Nanoliter 2010 injector (World Precision Instruments, Sarasota, FL, USA). For transcriptional analysis of *LsCDA1*, thirty individuals were randomly collected from each group at 4, 8, and 12 h post-injection. To further confirm whether *LsCDA1* was regulated by 20E synthesis and signaling, we performed RNAi experiments using dsRNAs of ecdysteroidogenesis genes (*CYP302a1*, *CYP306a1*, and *CYP314a1*) and 20E signal pathway genes (*E74*, *E78*, and *FTZ-F1*). These dsRNAs were prepared using a TranscriptAid T7 High Yield Transcription Kit (Thermo Scientific, Wilmington, DE, USA), and ds*GFP* was used as the negative control. The dsRNAs of each ecdysone-related gene were injected into fifth-instar larvae (300 ng/larva) using a microinjector. The larval samples were collected from each group at 3 days post-injection, and total RNA was extracted to analyze the RNAi efficiency and expression of *LsCDA1* by using qPCR as described above.

### 4.5. Function Analysis of LsCDA1 by RNAi

To evaluate the roles of *LsCDA1* in *L. serricorne* development, dsRNAs (300 ng) of *LsCDA1* and *GFP* were synthesized in vitro as described above and were injected into the fifth-instar larvae and the late pupae, respectively. The insects treated with dsRNA were reared under the same conditions as mentioned above. Insect survival and abnormal phenotypes were observed and photographed using a stereomicroscope (Keyence VHX-6000, Keyence Corporation, Osaka, Japan). To determine RNAi efficiency, the relative expression levels of *LsCDA1* in both the ds*LsCDA1* and ds*GFP* groups at 1, 3, and 5 days post-injection were detected by qPCR, as described above. To explore the effects of *LsCDA1* RNAi on chitin metabolism, a chitin content assay was performed according to the previously described method [69]. In brief, the chitin content of whole insect bodies was measured by the SpectraMax M2 microplate reader (Molecular Devices, Sunnyvale, CA, USA) at 3 days after dsRNA injection. The chitin contents were given as micrograms per milligram of insect body. After *LsCDA1* was knocked down in *L. serricorne*, the transcript levels of three chitin synthesis pathway genes (*LsTRE1*, *LsUAP1*, and *LsCHS1*) were detected by qPCR at 3 days after injection. To further explore the effects of *LsCDA1* RNAi on wing development, samples were collected from the late pupae injected with ds*LsCDA1* and ds*GFP* for 3 days. The relative expression levels of four wing development-related genes (*LsDY*, *LsWG*, *LsVG*, and *LsAP*) were determined by qPCR.

### 4.6. Statistical Analysis

Data were presented as mean ± standard error (SE) and were analyzed using SPSS 21.0 software (SPSS Inc., Chicago, IL, USA). Survival curves were analyzed using the Kaplan–Meier method, and the Log-Rank test was used to evaluate the significance of differences between the RNAi-treatment and control groups. Differences between two groups were compared using Student’s *t*-test (* *p* < 0.05, ** *p* < 0.01, *** *p* < 0.001). A one-way analysis of variance (ANOVA) followed by a least significant difference (LSD) test was applied for comparing the differences among more than two samples.

## 5. Conclusions

In summary, we identified a chitin deacetylase gene (*LsCDA1*) from *L. serricorne*. *LsCDA1* was highly expressed in the integument and showed periodic expression during each molting. The expression of *LsCDA1* could be regulated by a 20E pulse. The RNAi results reveal that *LsCDA1* plays vital roles in molting transitions by blocking cuticle chitin synthesis and deposition. The knockdown of *LsCDA1* inhibited the expression of wing development-related genes, resulting in wing deformities. These findings indicated *LsCDA1* as a potential target for the control of *L. serricorne*.

## Supplementary Materials

Supplementary materials can be found at https://www.mdpi.com/1422-0067/21/7/2449/s1, Table S1: Details of chitin deacetylase sequences used for phylogenetic analysis, Table S2: Primers used in this study.

## References

[1] Choi H.K., Choi K.H., Kramer K.J., Muthukrishnan S. (1997). Isolation and characterization of a genomic clone for the gene of an insect molting enzyme, chitinase. Insect Biochem. Mol. Biol..

[2] Merzendorfer H., Zimoch L. (2003). Chitin metabolism in insects: Structure, function and regulation of chitin synthases and chitinases. J. Exp. Biol..

[3] Moussian B., Schwarz H., Bartoszewski S., Nusslein-Volhard C. (2005). Involvement of chitin in exoskeleton morphogenesis in *Drosophila melanogaster*. J. Morphol..

[4] Nation J.L. (2008). Insect Physiology and Biochemistry.

[5] Cohen E. (2001). Chitin synthesis and inhibition: A revisit. Pest Manag. Sci..

[6] Kramer K., Muthukrishnan S. (2005). Chitin metabolism in insects. Compr. Mol. Insect Sci..

[7] Tetreau G., Cao X.L., Chen Y.R., Muthukrishnan S., Jiang H.B., Blissard G.W., Kanost M.R., Wang P. (2015). Overview of chitin metabolism enzymes in *Manduca sexta*: Identification, domain organization, phylogenetic analysis and gene expression. Insect Biochem. Mol. Biol..

[8] Xi Y., Pan P.L., Ye Y.X., Yu B., Xu H.J., Zhang C.X. (2015). Chitinase-like gene family in the brown planthopper, *Nilaparvata lugens*. Insect Mol. Biol..

[9] Xi Y., Pan P.L., Zhang C.X. (2015). The *β*-*N*-acetylhexosaminidase gene family in the brown planthopper, *Nilaparvata lugens*. Insect Mol. Biol..

[10] Xi Y., Pan P.L., Ye Y.X., Yu B., Zhang C.X. (2014). Chitin deacetylase family genes in the brown planthopper, *Nilaparvata lugens* (Hemiptera: Delphacidae). Insect Mol. Biol..

[11] Tsigos I., Martinou A., Kafetzopoulos D., Bouriotis V. (2000). Chitin deacetylases: New, versatile tools in biotechnology. Trends Biotechnol..

[12] Guo W., Li G., Pang Y., Wang P. (2005). A novel chitin binding protein identified from the peritrophic membrane of the cabbage looper, *Trichoplusia ni*. Insect Biochem. Mol. Biol..

[13] Arakane Y., Dixit R., Begum K., Park Y., Specht C.A., Merzendorfer H., Kramer K.J., Muthukrishnan S., Beeman R.W. (2009). Analysis of functions of the chitin deacetylase gene family in *Tribolium castaneum*. Insect Biochem. Mol. Biol..

[14] Yang W.J., Xu K.K., Yan X., Chen C.X., Cao Y., Meng Y.L., Li C. (2018). Functional characterization of chitin deacetylase 1 gene disrupting larval–pupal transition in the drugstore beetle using RNA interference. Comp. Biochem. Physiol. B.

[15] Wu J.J., Mu L.L., Chen Z.C., Fu K.Y., Guo W.C., Li C., Li G.Q. (2019). Disruption of ecdysis in *Leptinotarsa decemlineata* by knockdown of chitin deacetylase 1. J. Asia Pac. Entomol..

[16] Wu J.J., Chen Z.C., Wang Y.W., Fu K.Y., Guo W.C., Li G.Q. (2019). Silencing chitin deacetylase 2 impairs larval–pupal and pupal-adult molts in *Leptinotarsa decemlineata*. Insect Mol. Biol..

[17] Dixit R., Arakane Y., Specht C.A., Richard C., Kramer K.J., Beeman R.W., Muthukrishnan S. (2008). Domain organization and phylogenetic analysis of proteins from the chitin deacetylase gene family of *Tribolium castaneum* and three other species of insects. Insect Biochem. Mol. Biol..

[18] Ding G.W., Yu R.R., Yang M.L., Ma E.B., Yang J., Zhang J.Z. (2014). Molecular characterization and functional analysis of *chitin deacetylase 1* gene in *Oxya chinensis* (Orthoptera: Acrididae). Acta Entomol. Sin..

[19] Yu R.R., Liu W.M., Zhao X.M., Zhang M., Li D.Q., Zuber R., Ma E.B., Zhu K.Y., Moussian B., Zhang J.Z. (2019). *LmCDA1* organizes the cuticle by chitin deacetylation in *Locusta migratoria*. Insect Mol. Biol..

[20] Luschnig S., Batz T., Armbruster K., Krasnow M.A. (2006). *Serpentine* and *vermiform* encode matrix proteins with chitin binding and deacetylation domains that limit tracheal tube length in *Drosophila*. Curr. Biol..

[21] Liu S.H., Li H.F., Yang Y., Yang R.L., Yang W.J., Jiang H.B., Dou W., Smagghe G., Wang J.J. (2018). Genome-wide identification of chitinase and chitin deacetylase gene families in the oriental fruit fly, *Bactrocera dorsalis* (Hendel). Comp. Biochem. Physiol. D.

[22] Quan G., Ladd T., Duan J., Wen F., Doucet D., Cusson M., Krell P.J. (2013). Characterization of a spruce budworm chitin deacetylase gene: Stage- and tissue-specific expression, and inhibition using RNA interference. Insect Biochem. Mol. Biol..

[23] Han G., Li X., Zhang T., Zhu X., Li J. (2015). Cloning and tissue-specific expression of a chitin deacetylase gene from *Helicoverpa armigera* (Lepidoptera: Noctuidae) and its response to *Bacillus thuringiensis*. J. Insect Sci..

[24] Yan X.P., Zhao D., Zhang Y.K., Guo W., Wang W., Zhao K.L., Gao Y.J., Wang X.Y. (2018). Identification and characterization of *chitin deacetylase 2* from the American white moth, *Hyphantria cunea* (Drury). Gene.

[25] Zhang Z.Y., Yan J.M., Liu Q., Zhang Y.H., Gong J., Hou Y. (2019). Genome-wide analysis and hormone regulation of chitin deacetylases in silkworm. Int. J. Mol. Sci..

[26] Sandoval-Mojica A.F., Scharf M.E. (2016). Gut genes associated with the peritrophic matrix in *Reticulitermes flavipes* (Blattodea: Rhinotermitidae): Identification and characterization. Arch. Insect Biochem. Physiol..

[27] Wang C.Y., Cheng J., Lyu Z.H., Li Z.X., Chen J.X., Lin T. (2019). *Chitin deacetylase 1* and *2* are indispensable for larval–pupal and pupal–adult molts in *Heortia vitessoides* (Lepidoptera: Crambidae). Comp. Biochem. Physiol. B.

[28] Wang S., Jayaram S.J., Senti K., Tsarouhas V., Jin H., Samakovlis C. (2006). Septate-junction-dependent luminal deposition of chitin deacetylases restricts tube elongation in the *Drosophila* trachea. Curr. Biol..

[29] Gangishetti U., Veerkamp J., Bezdan D., Schwarz H., Lohmann I., Moussian B. (2012). The transcription factor grainy head and the steroid hormone ecdysone cooperate during differentiation of the skin of *Drosophila melanogaster*. Insect Mol. Biol..

[30] Zhang M., Ji Y.N., Zhang X.B., Ma P.J., Wang Y.W., Moussian B., Zhang J.Z. (2019). The putative chitin deacetylases *Serpentine* and *Vermiform* have non-redundant functions during *Drosophila* wing development. Insect Biochem. Mol. Biol..

[31] Toprak U., Baldwin D., Erlandson M., Gillott C., Hou X., Coutu C., Hegedus D.D. (2010). A chitin deacetylase and putative insect intestinal lipases are components of the *Mamestra configurata* (Lepidoptera: Noctuidae) peritrophic matrix. Insect Mol. Biol..

[32] Zhong X.W., Wang X.H., Tan X., Xia Q.Y., Xiang Z.H., Zhao P. (2014). Identification and molecular characterization of a chitin deacetylase from *Bombyx mori* peritrophic membrane. Int. J. Mol. Sci..

[33] Mahroof R.M., Phillips T.W. (2008). Life history parameters of *Lasioderma serricorne* (F.) as influenced by food sources. J. Stored Prod. Res..

[34] Li C., Li Z.Z., Cao Y., Zhou B., Zheng X.W. (2009). Partial characterization of stress-induced carboxylesterase from adults of *Stegobium paniceum* and *Lasioderma serricorne* (Coleoptera: Anobiidae) subjected to CO_2_-enriched atmosphere. J. Pest Sci..

[35] Minor M.F. (1979). Do adult cigarette beetle feed?. Tob. Sci..

[36] Riudavets J., Salas I., Pons M.J. (2007). Damage characteristics produced by insect pests in packaging film. J. Stored Prod. Res..

[37] Collins D., Conyers S. (2010). The effect of sub-zero temperatures on different lifestages of *Lasioderma serricorne* (F.) and *Ephestia elutella* (Hübner). J. Stored Prod. Res..

[38] Yu C., Subramanyam B., Flinn P.W., Gwirtz J.A. (2011). Susceptibility of *Lasioderma serricorne* (Coleoptera: Anobiidae) life stages to elevated temperatures used during structural heat treatments. J. Econ. Entomol..

[39] Imai T. (2015). The additive effect of carbon dioxide on mortality of the cigarette beetle *Lasioderma serricorne* (Coleoptera: Anobiidae) in low-oxygen atmospheres. Appl. Entomol. Zool..

[40] Saeed M.B.E.E.E.M., Laing M.D., Miller R.M., Bancole B. (2017). Ovicidal, larvicidal and insecticidal activity of strains of *Beauveria bassiana* (Balsamo) Vuillemin against the cigarette beetle, *Lasioderma serricorne* Fabricius (Coleoptera: Anobiidae), on rice grain. J. Stored Prod. Res..

[41] Hironaka M., Kamura T., Osada M., Sasaki R., Shinoda K., Hariyama T., Miyatake T. (2017). Adults of *Lasioderma serricorne* and *Stegobium paniceum* (Anobiidae: Coleoptera) are attracted to ultraviolet (UV) over blue light LEDs. J. Econ. Entomol..

[42] Rajendran S., Narasimhan K.S. (1994). Phosphine resistance in the cigarette beetle *Lasioderma serricorne* (Coleoptera: Anobiidae) and overcoming control failures during fumigation of stored tobacco. Int. J. Pest Manag..

[43] Sağlam Ö., Edde P.A., Phillips T.W. (2015). Resistance of *Lasioderma serricorne* (Coleoptera: Anobiidae) to fumigation with phosphine. J. Econ. Entomol..

[44] Abdelghany A.Y., Awadalla S.S., Abdel-Baky N.F., EL-Syrafi H.A., Fields P.G. (2016). Efficacy of reduced risk insecticides on penetration into jute and polyethylene bags by *Lasioderma serricorne* (F.) (Coleoptera: Anobiidae). J. Stored Prod. Res..

[45] Wu Y., Zhang W.J., Huang D.Y., Wang Y., Wei J.Y., Li Z.H., Sun J.S., Bai J.F., Tian Z.F., Wang P.J. (2015). Chemical compositions and insecticidal activities of *Alpinia kwangsiensis* essential oil against *Lasioderma serricorne*. Molecules.

[46] Cohen E. (1993). Chitin synthesis and degradation as targets for pesticide action. Arch. Insect Biochem. Physiol..

[47] Chen X.Y.L., Xu K.K., Yan X., Chen C.X., Cao Y., Wang Y.W., Li C., Yang W.J. (2018). Characterization of a *β*-*N*-acetylglucosaminidase gene and its involvement in the development of *Lasioderma serricorne* (Fabricius). J. Stored Prod. Res..

[48] Yang W.J., Xu K.K., Yan Y., Li C. (2019). Knockdown of *β*-*N*-acetylglucosaminidase *2* impairs molting and wing development in *Lasioderma serricorne* (Fabricius). Insects.

[49] Blair D.E., Schuttelkopf A.W., MacRae J.I., vanAalten D.M. (2005). Structure and metal-dependent mechanism of peptidoglycan deacetylase, a streptococcal virulence factor. Proc. Natl. Acad. Sci. USA.

[50] Yu H.Z., Liu M.H., Wang X.Y., Yang X., Wang W.L., Geng L., Yu D., Liu X.L., Xu J.P. (2016). Identification and expression profiles of chitin deacetylase genes in the rice leaf folder, *Cnaphalocrocis medinalis*. J. Asia Pac. Entomol..

[51] Luan J.B., Ghanim M., Liu S.S., Czosnek H. (2013). Silencing the ecdysone synthesis and signaling pathway genes disrupts nymphal development in the whitefly. Insect Biochem. Mol. Biol..

[52] Niwa R., Niwa Y.S. (2014). Enzymes for ecdysteroid biosynthesis: Their biological functions in insects and beyond. Biosci. Biotechnol. Biochem..

[53] Hill R.J., Billas I.M., Bonneton F., Graham L.D., Lawrence M.C. (2013). Ecdysone receptors: From the Ashburner model to structural biology. Annu. Rev. Entomol..

[54] Yao Q., Zhang D.W., Tang B., Chen J., Chen J., Lu L., Zhang W.Q. (2010). Identification of 20-hydroxyecdysone late-response genes in the chitin biosynthesis pathway. PLoS ONE.

[55] Li D.Q., Zhang J.Q., Wang Y., Liu X.J., Ma E.B., Sun Y., Li S., Zhu K.Y., Zhang J.Z. (2015). Two chitinase 5 genes from *Locusta migratoria*: Molecular characteristics and functional differentiation. Insect Biochem. Mol. Biol..

[56] Rong S., Li D.Q., Zhang X.Y., Li S., Zhu K.Y., Guo Y.P., Ma E.B., Zhang J.Z. (2013). RNA interference to reveal roles of *β*-*N*-acetylglucosaminidase gene during molting process in *Locusta migratoria*. Insect Sci..

[57] Yang W.J., Xu K.K., Cong L., Wang J.J. (2013). Identification, mRNA expression, and functional analysis of chitin synthase 1 gene and its two alternative splicing variants in oriental fruit fly, *Bactrocera dorsalis*. Int. J. Biol. Sci..

[58] Zhuo W.W., Fang Y., Kong L.F., Li X., Sima Y.H., Xu S.Q. (2014). Chitin synthase A: A novel epidermal development regulation gene in the larvae of *Bombyx mori*. Mol. Biol. Rep..

[59] Shi J.F., Mu L.L., Guo W.C., Li G.Q. (2016). Identification and hormone induction of putative chitin synthase genes and splice variants in *Leptinotarsa decemlineata* (SAY). Arch. Insect Biochem. Physiol..

[60] Lyu Z.H., Chen J.X., Li Z.X., Cheng J., Wang C.Y., Lin T. (2019). Knockdown of *β*-*N*-acetylglucosaminidase gene disrupts molting process in *Heortia vitessoides* Moore. Arch. Insect Biochem. Physiol..

[61] Chen C., Yang H., Tang B., Yang W.J., Jin D.C. (2017). Identification and functional analysis of chitinase 7 gene in white-backed planthopper, *Sogatella furcifera*. Comp. Biochem. Physiol. B.

[62] Clark-Hachtel C.M., Linz D.M., Tomoyasu Y. (2013). Insights into insect wing origin provided by functional analysis of vestigial in the red flour beetle, *Tribolium castaneum*. Proc. Natl. Acad. Sci. USA.

[63] Li C., Li B., Ma S., Lü P., Chen K. (2017). Dusky works upstream of Four-jointed and Forked in wing morphogenesis in *Tribolium castaneum*. Insect Mol. Biol..

[64] Larkin M.A., Blackshields G., Brown N.P., Chenna R., McGettigan P.A., McWilliam H., Valentin F., Wallace I.M., Wilm A., Lopez R. (2007). Clustal W and Clustal X version 2.0. Bioinformatics.

[65] Kumar S., Stecher G., Tamura K. (2016). MEGA7: Molecular evolutionary genetics analysis version 7.0 for bigger datasets. Mol. Biol. Evol..

[66] Yang W.J., Xu K.K., Cao Y., Meng Y.L., Liu Y., Li C. (2019). Identification and expression analysis of four small heat shock protein genes in cigarette beetle, *Lasioderma serricorne* (Fabricius). Insects.

[67] Livak K.J., Schmittgen T.D. (2001). Analysis of relative gene expression data using real-time quantitative PCR and the 2^-ΔΔCT^ method. Methods.

[68] Bustin S.A., Benes V., Garson J.A., Hellemas J., Huggett J., Kubista M., Mueller R., Nolan T., Pfaffl M.W., Shipley G.L. (2009). The MIQE guidelines: Minimum information for publication of quantitative real-time PCR experiments. Clin Chem..

[69] Yang W.J., Chen C.X., Yan Y., Xu K.K., Li C. (2020). Clip-domain serine protease gene (*LsCLIP3*) is essential for larval–pupal molting and immunity in *Lasioderma serricorne*. Front. Physiol..

