# Supplementary material for: Role of Chitin Deacetylase 1 in the Molting and Metamorphosis of the Cigarette Beetle Lasioderma serricorne
Source: Int J Mol Sci. 2020 Apr 1;21(7):2449. doi: 10.3390/ijms21072449 (PMC7177437; doi:10.3390/ijms21072449)
Supplement: Supplementary file 1 [file ijms-21-02449-s001.pdf]

Supplementary Tables

Table S1. Details of chitin deacetylase sequences used for phylogenetic analysis.

Insect Species	Gene Name	Accession Number
Bombyx mori	*BmCDA1*	XP_004929283.1
	BmCDA2A	NP_001103795.1
	BmCDA2B	NP_001103796.1
	BmCDA3A	XP_021207356.1
	BmCDA3B	XP_004931841.2
	BmCDA4	XP_012548585.1
	BmCDA5	XP_021207767.1
	BmCDA6	XP_004923454.1
	BmCDA7	XP_004923480.1
	BmCDA8	XP_004923455.1
Bactrocera dorsalis	*BdCDA1*	AUP42574.1
	BdCDA2A	AUP42576.1
	BdCDA2B	AUP42575.1
	BdCDA3	AUP42577.1
	BdCDA4	AUP42578.1
	BdCDA5	XP_011210019.1
	BdCDA9	AUP42579.1
Drosophila melanogaster	*DmCDA1*	NP_730444
	DmCDA2	NP_001163469
	DmCDA3	NP_609806
	DmCDA4	NP_728468
	DmCDA5	NP_001097044
	DmCDA9	NP_611192
Apis mellifera	*AmCDA1*	XP_391915
	AmCDA2	XP_623723
	AmCDA4	XP_001120478
	AmCDA5	XP_624655
Tribolium castaneum	*TcCDA1*	NP_001095946
	TcCDA2A	NP_001096047
	TcCDA2B	NP_001116303
	TcCDA3	NP_001104011
	TcCDA4	NP_001103903
	TcCDA5A	NP_001103739
	TcCDA5B	NP_001107799
	TcCDA6	NP_001103905
	TcCDA7	NP_001104012
	TcCDA8	NP_001103906
	TcCDA9	NP_001103904
Nasonia vitripennis	*NvCDA1*	XP_001604765
	NvCDA2	XP_001604838
	NvCDA3	XP_001606617
	NvCDA4	XP_001607989
	NvCDA5	XP_001603918
Lasioderma serricorne	*LsCDA1*	AXU05961.1
Mamestra brassicae	*MbCDA1*	AEI30868.1

Stegobium paniceum	*SpCDA1*	AYA83838.1
Helicoverpa armigera	*HaCDA1*	ADB43610.1
	HaCDA2	KM598637
Heortia vitessoides	*HvCDA1*	MK389498
	HvCDA2	MK389499
Locusta migratoria	*LmCDA1*	ANA57443.1
	LmCDA2A	ANA57444.1
	LmCDA2B	ANA57445.1

Table S2. Primers used in this study.

Application of Primers	Gene Name	Forward Primer (5′-3′)	Reverse Primer (5′-3′)
ORF confirmation	*LsCDA1*	ATTTGTGATGGCGCCACGTTTA	TTAGAAGAAACCATCTCCGGTGG
qPCR analysis	*LsCDA1*	GCTTTCTTGTTTTGGGTGGA	CGAGGTCAATTTGCAGGAAT
	LsTRE1	CCTACGGACCAAGACCTGAA	TTTGTTGGTTGCGTTGGTAA
	LsUAP1	GGCAACACTGATCCATCCTT	GGCACTCTGCACGATTATGA
	LsCHS1	AAAGAAGAATCTGGTTCTCT	ATATAGTGCTCTCGTCACCA
	LsDY	GAGATGACGGACGTGAACAC	TCAACACCAACAGCAGCATC
	LsWG	AGCCTCCCCAATCCCACT	AGGGCAGCATCGAGACCT
	LsVG	GGGCTGTTCACGATTACCAT	GAATCCTGAACTTGCGCTTC
	LsAP	TTTTCGGAATGAGGAGATGC	AAGGCGATGGATAATGCAAG
	EF1α	GCATCTCCACGGATTTCACT	AAGGCAAGACGCTTATCGAA
	18S	GTTGATCACGTCGCAAGCTA	AGGTTTCCCTCTGGCTTGTT
dsRNA synthesis	*LsCDA1*	TAATACGACTCACTATAGGGACCTCTGTTGTATAC TGATG	TAATACGACTCACTATAGGGCGTTCATCATCATTG TGAGT
	LsCYP302a1	TAATACGACTCACTATAGGGTTTGATCGTCTACAC CAGAA	TAATACGACTCACTATAGGGAACTGCTCATTGCTT CTACT
	LsCYP306a1	TAATACGACTCACTATAGGGATCGTCGGTTACTTG CCTTT	TAATACGACTCACTATAGGGGCGCTATGCTTGCTC ATGCC
	LsCYP314a1	TAATACGACTCACTATAGGGAAGCATATAAGCGG TTGGTT	TAATACGACTCACTATAGGGGGTACCAACAAGAA CTCAAA
	LsE74	TAATACGACTCACTATAGGGCGCCGCCTCCGACT AAACATC	TAATACGACTCACTATAGGGGGTATGAGTGGTGC CATCTGT
	LsE78	TAATACGACTCACTATAGGGCGACCAGTCCGATT CCGAAAC	TAATACGACTCACTATAGGGCGGCACCCTCTTGG CAAACTC
	LsFTZ-F1	TAATACGACTCACTATAGGGCGAAAATATGTCATT GTTT	TAATACGACTCACTATAGGGAGTGCTCGTCGAAC TGATTCG
	GFP	TAATACGACTCACTATAGGGCAGTTCTTGTTGAAT TAGATG	TAATACGACTCACTATAGGGAATGTTACCATCTTC TTTAA
